# Current Trends in Nanomaterial-Based Amperometric Biosensors

**DOI:** 10.3390/s141223439

**Published:** 2014-12-08

**Authors:** Akhtar Hayat, Gaëlle Catanante, Jean Louis Marty

**Affiliations:** 1 BIOMEM, Universitéde Perpignan, 52 Avenue Paul Alduy, 66860 Perpignan Cedex, France; E-Mails: akhtarloona@gmail.com (A.H.); gaelle.catanante@univ-perp.fr (G.C.); 2 Interdisciplinary Research Centre in Biomedical Materials (IRCBM), COMSATS Institute of Information Technology (CIIT), Lahore 54000, Pakistan

**Keywords:** electrochemical sensing, amperometric biosensors, nanomaterials, sensing design, analytical applications

## Abstract

The last decade has witnessed an intensive research effort in the field of electrochemical sensors, with a particular focus on the design of amperometric biosensors for diverse analytical applications. In this context, nanomaterial integration in the construction of amperometric biosensors may constitute one of the most exciting approaches. The attractive properties of nanomaterials have paved the way for the design of a wide variety of biosensors based on various electrochemical detection methods to enhance the analytical characteristics. However, most of these nanostructured materials are not explored in the design of amperometric biosensors. This review aims to provide insight into the diverse properties of nanomaterials that can be possibly explored in the construction of amperometric biosensors.

## 1. Introduction

Biosensors have emerged as an alternative tool to classical chromatographic methods for the analysis of analyte samples. A biosensor functions by converting some biological response into a detectable signal. The commonly used biomolecules in biosensor design include DNA/RNA, antibodies, enzyme, peptides, microorganisms and whole cells. The signals obtained are processed and transferred to a display or data storage device [1,2]. The main advantageous characteristic of a biosensor is the direct spatial contact between the bio-recognition element and the transducer surface [3]. The output signal of biosensor can be measured with electrochemical, quartz crystal microbalance, optical absorption fluorescence, surface plasmon resonance, radioactive, magnetic and other transducer platforms. Among all these transduction methods, electrochemistry provides an attractive way of encompassing the advantages of instrumental simplicity, moderate cost, and portability for decentralized analysis. The modern and newly emerged electrochemical methods are sensitive, selective, facile and rapid making them suitable candidate for diverse analytical applications. A number of electrochemical methodologies have been explored in the field of electrochemical biosensors such as differential pulse voltammetry, cyclic voltammetry, linear sweep voltammetry, square wave voltammetry, amperometry and impedimetric analysis [4,5]. Amperometry is the common most electrochemical technique which has been widely employed in the domain of electrochemical biosensors. Amperometry measures the amplitude of a reduction or oxidation current at a given specific potential over a fixed period of time. Despite much progress, increased interest in the development of new materials applicable in amperometric techniques to fulfill the necessity of control specific biomolecular interaction still exists. Important advances have been achieved with the utilization of nanomaterials in the design of electrochemical biosensors to improve their analytical performance [6].

## 2. Functions of Nanomaterials

The last decade has witnessed an enormous impact of nanomaterials on the field of biosensors [7]. Significant progress has been achieved in synthetic approaches to prepare nanomaterials of desired properties such as controllable size, shape, surface charge and physiochemical characteristics [8,9]. Nanomaterials can be decorated with polymers and biomolecules to achieve better biocompatibility and precise targeting. These features have made it possible to integrate nanomaterials in biosensors for any desired function, and led to an increasing use of nanomaterials in the design of electrochemical amperometric sensors. Thousands of scientific articles describing associations between nanomaterials and biomolecules have been published in the literature [10]. In this context, different techniques have been explored to integrate nanomaterials of diverse nature in the electrochemical transduction platform. The Langmuir-Blodgett method was the pioneering approach in the fabrication of thin films formed by transferring an amphiphilic materials dispersed at air/water interface onto a solid substrate [11]. Another interesting method providing high control of thickness at the nanoscale level was developed by Decher [12]. Despite the specific chemical interactions, the proposed layer by layer methodologies rely on the coulombic electrostatic that provides multilayer growth. This strategy has been proven by various researchers as the simplest tool for film fabrication which can be applied to various types of materials like polyelectrolytes [13], metallophthalocyanines [14], carbon nanotubes [15] and all types of nanoparticles [16]. Recent trends have also shown the direct conjugation of nanomaterials with biomolecules or target analyte through various kinds of interactions such as physical adsorption, covalent attachment and electrostatic interaction with week hydrogen bonding. However, in some cases nanomaterials are directly employed in the interacting medium on transducer surface to enhance the electroanalytical performance of the system [17]. There is no doubt on the importance of nanostructured materials and their implications in biosensor fabrication. In this review paper, we discuss recent trends in the field of amperometric biosensors exploring the diverse functions of nanomaterials to improve the analytical figures of the biosensors.

**Figure 1.**
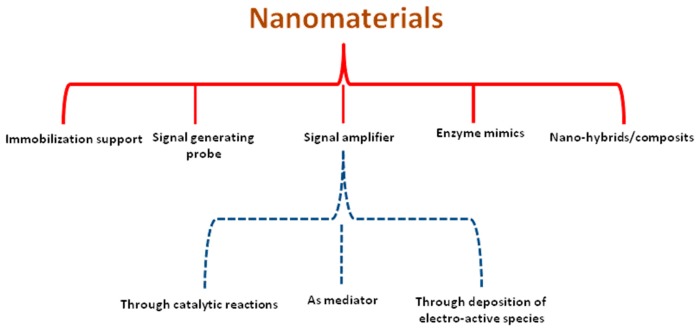
Possible functions that can be achieved with nanomaterial integration in amperometric biosensors.

Nanotechnolgy is defined as the creation of functional systems through control of matter at the 1–100 nm scale. Nanomaterials exhibit interesting physical, chemical, and electronic properties such as size and shape dependent surface plasmon resonance, high surface energy and surface to volume ratio and tunable surface properties [18,19]. For example, the physical properties of nanomaterials can be explored for cell imaging, drug delivery systems and to design optical assays and bioassays. Similarly, the chemical properties of the nanomaterials facilitate nano surface modification with a variety of functional groups to attach molecules of diverse nature. The integration of the electronic properties of nanomaterials has dramatically reduced the size of electronic devices. In the same context, these intrinsic and distinguished features make nanomaterials the best fit for designing novel biosensor platforms. A wide variety of nanomaterials including metal nanoparticles, oxide nanoparticles, quantum dots, nanowires, nanoshells, carbon nanotubes and even composite nanoparticles have found attractive applications in many kind of amperometric biosensors for a broad spectrum of analytical applications. Nanomaterials of different nature, size, shape and composition can play various roles in different sensing systems [20]. However, the organization of nanomaterials into controlled surface architectures is essential for the successful realization of these properties. The important possible functions that can be achieved with nanomaterial integration are listed below, presented in Figure 1, and will be discussed in detail in the next sections.

(1) Immobilization platform for biomolecules
(2) Amperometric signal amplification
(3) Nanomaterials as signal generating probe
(4) Nanomaterials as enzyme mimics
(5) Hybrid and composite nanomaterials in amperometric biosensors

### 2.1. Immobilization Platform for Biomolecules

**Figure 2.**
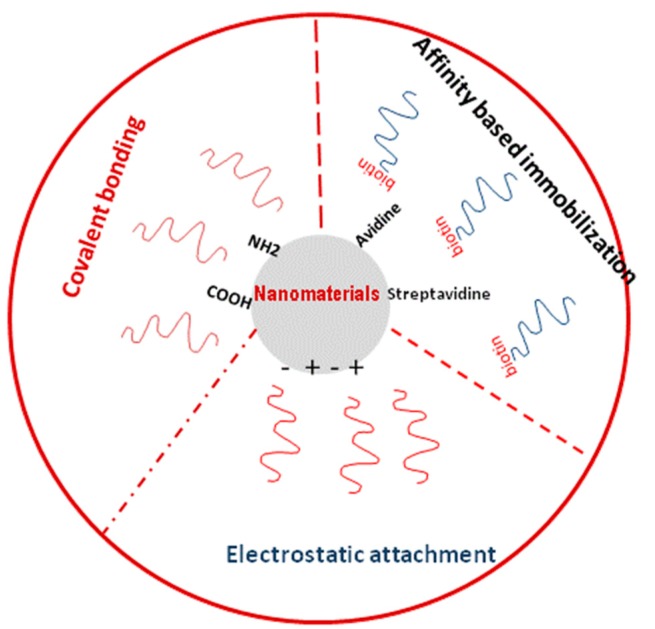
Nanomaterial-based immobilization methodologies.

Recent interest in nanotechnology has provided a wealth of diverse nanoscaffolds that could be used to support biomolecules/analyte immobilization due to their potential features (Figure 2) [21,22]. Immobilization of biomolecules is an advantageous step towards the commercialization of amperometric sensors due to the possible resulting increase in physiological stability. The premise of employing nanomaterials for immobilization on a transducer surface is to reduce diffusion limits and maximize the surface area to increase biomolecule loading [23]. In the last years several types of nanomaterials have been produced in various nanostructure shapes like nanorods, nanowires, nanotubes and nanorings for immobilization purposes [24,25]. Nanomaterials have a strong tendency to adsorb biomolecules and to help the immobilization of biomolecules in biosensor construction. The adsorption of biomolecules directly onto bulk materials may result in denaturation and loss of bioactivity, while nanosize materials retain the bioactivity of biomolecules. It is of vital importance that several nanoparticles carry charges and hence they can provide electrostatic surface to attach the biomolecules with different charges.

Among all the nanomaterials, magnetic nanoparticles have been used extensively to immobilize both biomolecules and analytes on the transducer surface [26–28]. Several types of magnetic nanoparticles and magnetic supports such as microspheres of diverse biomaterials encapsulating the magnetic nanoparticles and copolymers with magnetic nanoparticles have been employed in biosensor fabrication with good results [29,30]. The high surface to volume ratio of magnetic nanoparticles facilitates high binding capacity and high catalytic specificity of the conjugated biomolecules. Magnetic nanoparticles decorated with streptavidin, and having amine and carboxylic functional groups to immobilize specific modified biomolecules/analytes are commercially available [31–33]. Similarly protein G magnetic beads to specifically immobilize antibodies are available [34]. The magnetic beads-based immobilization methodology is easy to operate with simple application of a magnet to provide a magnetic field to capture modified the modified nanoparticles on the transducer surface [35,36]. However, the application of magnetic-based methods is restricted due to lack of reproducibility and renewal of the sensor surface. Future work in this regard may focus on improving the pitfalls of magnetic nanoparticles used by designing new strategies. For example, due to the ease of manipulation and separation using a magnetic field, it is possible to simply ‘switch off’ the magnetic effects by removing the magnetic induction field, which can be advantageous for bead recovery. Moreover, magnetic beads can be manipulated independent of normal microfluidic or biological processes which results in improved exposure of the functionalized bead surface to the surrounding viscous liquid, due to the increased relative motion of the bead with respect to the fluid. Additionally, the application of a revolving external magnetic during manipulation could also be beneficial to disperse the magnetic beads uniformly on the reactor surface to obtain reproducible sensing surfaces. Besides magnetic nanoparticles, gold nanoparticles serve as an excellent platform for the immobilization of biomolecules since the interaction between amino and cysteine groups of biomolecules can be explored apart from gold and thiol chemistry [37–39]. Several workers have reported a one-step method for immobilization of biomolecues on transducer surfaces through the integration of poly(methyl methacrylate) nanoparticles [40]. Non-metallic polystyrene nanoparticles have also been used in the literature to improve the biomolecule immobilization efficiency [23]. Nanoparticles of silver, silica, zinc oxide, aluminium oxide, zirconium oxide, manganese oxide and nickel oxide were explored to immobilize biomolecules, specifically enzymes, to construct amperometric biosensors [41–44]. Recent research has focused on the polymerization of different molecules on the nanoparticles, and subsequently using them as anchoring supports in the fabrication of biosensors. Martin *et al*., prepared novel core–shell Fe_3_O_4_@poly(dopamine) magnetic nanoparticles through an *in situ* self-polymerization method. The core–shell nanoparticles were employed as solid supports for the covalent immobilization of horseradish peroxidase (HRP), and the resulting biofunctionalized magnetic nanoparticles were employed to construct an amperometric biosensor for H_2_O_2_. The enzyme biosensor showed a high sensitivity, a low limit of detection, a wide linear range and high stability for 1 month [29]. Lu *et al*., proposed a novel, high-yield and template-free method for the synthesis of Ag nanoparticle-decorated thionine/infinite coordination polymer (AgNP/THI/ICP) fibers. The thionine was adsorbed to the AgNP/THI/ICP fibers by π-conjugation and acted as the redox probe. The AgNP/THI/ICP fibers not only favored the antibody immobilization but also facilitated the electron transfer [45]. Turkmen *et al*., fabricated a new biosensor based on immobilization of glucose oxidase (Gox) using an enzyme solution containing *o*-phenylenediamine(oPD) on platinum nanoparticles (PtNPs) electrodeposited polyvinylferrocenium perchlorate matrix (PVF+ClO_4_^−^). Gox was immobilized simultaneously with the electropolymerization of oPD on PtNPs/PVF+ClO_4_^−^/Pt, and the resulting biosensor showed excellent anti-interference ability to ascorbic acid and uric acid [46]. Despite the much progress in nanomaterials integration in electrochemical biosensors, some of the above described nanomethods are yet to be explored in the construction of amperometric biosensors.

Recent research to explore the hidden properties of nanomaterials has indicated that most nano-materials exhibit intrinsic enzyme-like properties that can be used to construct enzyme-free sensors to detect target analytes. This reactive nature of nanomaterials can be advantageous in one way, but on the other hand, it may limit the applications of nanomaterials as immobilization supports. One such example is the construction of a glucose biosensor based on the reactivity of ferromagnetic nanoparticles towards hydrogen peroxide [47]. This indicates that use of nanomaterials as immobilization support may increase the non-specific signal and yield irreproducible results due to their reactivity with commonly used optical and electrochemical signal-generating probes. To control the surface reactivity, different strategies, including use of surfactants, encapsulation of the nanomaterial surface and variation in pH have been employed in recent years. Based on these findings, it will be of vital importance and interest to use inert nanomaterials in the construction of biosensors to overcome the problem of non-specific reactivity.

### 2.2. Amperometric Signal Amplification

**Figure 3.**
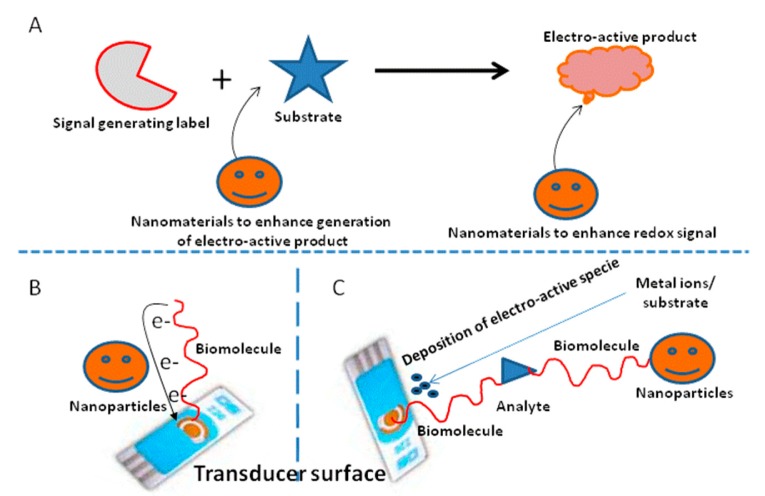
Nanomaterial-based signal amplification through (**A**) catalytic properties; (**B**) mediator; (**C**) deposition of electrochemically active species.

The detection of ultralow analyte concentrations is required not only in the chemical laboratory, but also in variety of areas including clinical diagnostic, food safety and environmental protection. Analytical figures of merits are continuously being pushed down by the gradual improvement of analytical technologies. The sensitivity of any protocol is related to the correlation between the analyte concentration and the strength of the output signal. Many efforts have been devoted to the exploration of novel means to realize ultrasensitive detection. Such strategies include the use of new labels (electroactive molecules, redox complexes, and metal ions), polymerase chain reaction, mass spectrometery and the integration of enzyme-assisted signal amplification processes [17]. Although these methods have increased the sensitivity, they are destructive and mainly suffer from time-consuming derivatization, high cost and the need for professional operation. With the introduction of nanotechnology and nanoscience, nanomaterial-based signal amplification has attained great importance in realizing high sensitivity and selectivity for the detection of analytes due to the rapid analysis procedures and easy miniaturization. A lot of nanomaterials including metal nanoparticles, semiconductor nanoparticles and carbon nanosized structures have emerged as electrochemical signal amplifiers in the design of electrochemical devices [48].The possible mechanisms for nanomaterial signal amplification are depicted in the Figure 3.

#### 2.2.1. Signal Amplification through Catalytic Reaction

The combination of the catalytic properties of nanomaterials with the unique properties of biosensors can results in the fabrication of highly sensitive sensors. Bio-functionalized nanoparticles can produce synergistic effects between catalytic activity to accelerate electrochemical signals, leading to lower and lower detection limits [49], even reaching zeptomolar concentrations. Furthermore, the linear correlation ranges becomes wider and wider. For example, carboxy-modified graphene oxide has been shown as a catalytic material for the oxidation of hydrogen peroxide [50], and carboxy-modified electrodes may have a synergistic effect towards the oxidation of hydrogen peroxide with subsequent improvement in the sensitivity of the method [51]. Platinum nanoparticles also exhibit catalytic properties and have been used in the fabrication of electrochemical sensors [52,53]. The platinum modified electrodes showed sensitive responses to H_2_O_2_ and lowered the oxidation potential. Since H_2_O_2_ is the product of many enzymatic reactions, the proposed platinum modified electrodes may find potential application in electrochemical biosensors for many substances. Similarly Ni nanoparticle-modified electrodes have been shown to possess electrocatalytic activity in the electro-oxidation of sugars, including glucose, sucrose, fructose and lactose [54]. The application of copper nanoparticle-modified transducer surfaces for sensitive detection of amino acids has been demonstrated [55]. Copper oxide nanoparticle-modified electrode surfaceshave shown catalytic properties in the detection of amikacin in another study [56]. Based on their redox properties, nanoceria-modified electrode surfaces have been employed to improve the analytical performance of electrochemical biosensors for a variety of target analytes [49,57].Electrochemical biosensors based on the catalytic properties of other nanoparticles have also been reported in the literature, for example silver and gold nanoparticles have been used in the construction of electrochemical biosensors for the detection of glucose [58,59].Fe_2_O_3_-, silica-, Pd-, ZnO- and MnO_2_-modified electrodes have been explored for this purpose. Apart from the nanomaterials described above, carbon-based materials such as single walled and multiwalled carbon nanotubes have been also used to generate electrochemical signals [60]. For example, Yang *et al*., fabricated a sandwich-type amperometric immunosensor for carcinoembryonic antigen by using functionalized carbon nanotubes decorated with concanavalin A along with horseradish peroxidase-labeled carcinoembryonic secondary antibodies. The HRP-Ab2 was assembled onto carbon nanotubes templates and the carcinoembryonic antibody was immobilized on the gold electrode modified by a cysteine monolayer. The developed amperometric immunosensor showed excellent analytical characteristics in terms of sensitivity and linear range [61]. Some of these reported materials are not investigated in the construction of amperometric biosensors, so future work in the domain of amperometric biosensors may focus on this.

#### 2.2.2. Nanomaterials as Mediator

Electrical contact between redox biomolecules and electrode surface is of vital importance in the construction of electrochemical sensors. However, biomolecules have no direct communication with the electrode surface because the active sites of biomolecules are surrounded by considerably thick insulating protein shells and the electron transfer between the two moieties is blocked, posing a problem for the electrochemical measurements. In this context, the conductive properties of the metallic nanoparticles to enhance the electron transfer rate between the biomolecules and the transducer surface are very well established. Metallic nanoparticles play the role of electron transfer mediators or electrical wires to replace the commonly used mediator in the construction of electrochemical biosensors. Although, the role of metallic nanoparticles as electron transfer mediators has been extensively explored for amperometric biosensors, however, some non-metallic nanoparticles such as oxide nanoparticles and semiconductor nanoparticles can also improve the electron transfer rate between proteins and electrodes. The improvement in electron flow is related to the conductivity of nanoparticles and arrangement between nanoparticles and biomolecules. The creation of well defined and ordered arrangments of nanoparticles using advanced and novel nano-based methods is a promising approach for the fabrication of amperometric biosensors with enhanced electron transfer properties. Gold nanoparticles have been used to improve the electron transfer between biomolecules and electrode surfaces in the construction of an enzymatic biosensor [62]. A seven-fold faster response was observed in the presence of nanoparticles as compared to the enzyme signal with its natural substrate [63]. Gold nanoparticles have also been integrated in the construction of redox protein-based amperometric biosensors to improve the electron transfer rate. Besides gold nanoparticles, silver nanoparticles have also good electrical conductivity and hence can be used to enhance the electron transfer between biomolecules and the transducer surface. Silver nanoparticles in conjugation with pyrolytic graphite electrodes have been reported to act as electrical bridges that wire the electron transfer between biomolecules and the electrode surface [64]. In the same context, Jimenez *et al*. investigated the electrocatalytic performance of electrodes modified with Pt nanoparticles (PtNPs) and two dendritic hyperbranched carbosilane polymers for the NADH oxidation. The proposed strategy permitted them to develop efficient biosensors capable of measuring NADH from +0.3 V (*vs*. SCE) providing a total protection *vs*. the poisoning of the electrodes. Based on these interesting and useful characteristics, the authors constructed an amperometric alcohol biosensor with alcohol dehydrogenase (ADH) [65].

#### 2.2.3. Nanomaterials as Seeds/Mediators to Deposit Electrochemically Active Species and Electrocatalysts

The nanomaterial-modified electrode surfaces can be used as seeds to initiate the deposition of metals through a seed-mediated nucleation or growth mechanism [66,67]. The modifying medium consists of metal salts and a reducing agent, and nanoparticles on the electrode surface serve as nucleation sites to facilitate the metal deposition, which results in higher loading of electroactive species and subsequently improves the nanoparticles-transducer surface communication. This improvement can be attributed to the fact that the deposited nanoparticles are closer to the electrode surface as compared to the nanoparticle labels. Such a feature is of vital importance in the case of competitive and labeled electrochemical immuno- and aptasensors [68], where the distance between the signal label and the electrode surface is too large for an efficient electron transfer due to the large size of both the analyte and the biomolecules. Additionally, the freshly seed-mediated nanoparticles are more electroactive, since they are free from any stabilizer, making them a highly attractive and useful tool in the design of affinity-based amperometric biosensors. This method has been employed to deposit electrochemically active metallic species including silver, copper and gold from their salts using a reducing agent for signal amplification [69–71]. So far, an enormous number of electrochemical biosensors based on this methods with developer solutions has been reported in the literature [48].

In another methodology, the seed approach is employed to deposit electrocatalysts. For the detection of an analyte, the transducer surface is incubated in a solution containing a high concentration of substrate which can be electrochemically oxidized or reduced by the electrocatalyst. The concentration of the deposited electrcatalyst can be indirectly measured on the basis of the catalytic current resulting from electrocatalysis of a substrate [72,73]. Since a clean surface can provide efficient binding of substrates, the catalytic properties of these particle-modified electrode surfaces is expected to be very high. In this concept, Pt has been shown to bean excellent catalyst for electrocatalysis of a range of substrates such as hydrazine, protons and H_2_O_2_. Therefore Pt is an ideal for signal amplification in the design of electrochemical amperometric biosensors for the above described compounds based on this strategy, if a suitable developer solution is available [74,75]. However, the advantages of seed-mediated deposition of Pt remain relatively unexplored, due to the difficulty in finding a suitable and an appropriate developer solution. In this context, some efforts have been made to explored different developer solutions [76], but an ideal solution for signal amplification employing this strategy is still not available.

Nanomaterials have a tendency to strongly adsorb on different supports. Nanoparticles may carry negative or positive zeta potential values, making their surfaces negative or positive. The presence of such a charge further enhances the non-specific adsorption of nanomaterials due to electrostatic interactions. The non-specific adsorption of nanomaterials surface may impact the generated signals in three ways: (a) by blocking the surface and generating electron transfer resistance to the flow of current which may result in a shift of redox potentials to higher values, increasing the probability of interfering signals due to elevated redox potentials; (b) the charged surface of nanomaterials may increase or decrease the rate of flow of electrons depending on the charge; (c)the reactivity associated with the non-specifically adsorbed nanoparticles may also impact the obtained signal. Compared to nanomaterials, it is very easy and simply to control the non-specific adsorption of biomolecules through the use of different proteins. However, use of these blocking proteins is not so fruitful in the case of nanomaterials. Future research in the field may focus on overcoming the problem of non-specific adsorption of nanomaterials on the transducer surface. Similarly, the other big challenge with the re-usability of nanomaterials may hinder their application in amperometric biosensors, and consequently future research may focus on this issue to solve the problem. However, based on the intrinsic physical and chemical properties of nanomaterials, it is expected that they will provide building blocks for the construction of amperometric biosensors to improve the analytical performance of the system.

### 2.3. Nanomaterials as Signal Generating Probes

**Figure 4.**
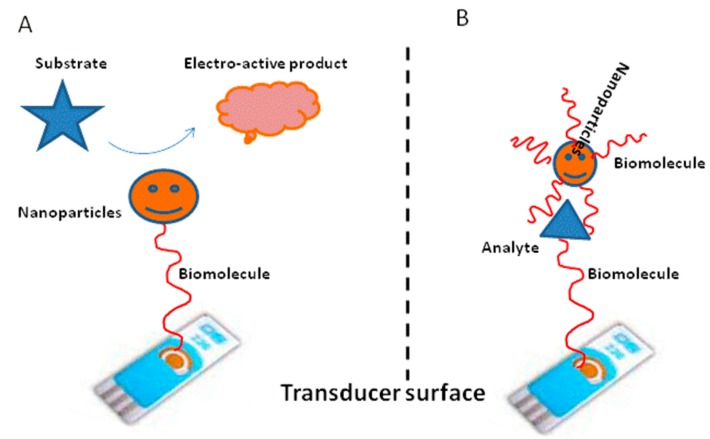
Nanomaterials as labels: (**A**) Direct assays; (**B**) Sandwich type assays.

Different approaches have been employed to obtain the electrochemical output signal in the construction of biosensors (Figure 4). Most conventional approaches involve the use of labels such as enzymes, electroactive molecules, redox complexes and metal ions. The emergence of nanotechnology has opened new horizons for the use of nanomaterial labels to obtain the electrochemical signal in the biosensors. The power and scope of such nanomaterials can be greatly enhanced by modifying the target analyte or biomolecules with these particles. Biomolecules labeled with nanomaterials retain their bioactivity and indentify their counterparts. The enormous signal enhancement associated with the use of nanomaterial labels provides the basis of ultra-sensitive affinity-based electrochemical biosensors. The strategies which utilize nanoparticles as labels can be sub-classified into two main groups: (a) nanomaterials that increase the loading of electroactive species; (b) nanoparticles that act as ultramicroelectrode arrays for the electrolysis of large amounts of substrates. Among the many kind of nanomaterials, gold nanoparticles are the most frequently used labels in electrochemical biosensors. Semiconductor and oxide nanoparticles are also available as labels to replace the commonly used enzyme labels in bioaffinity biosensors. Immuno- and aptasensors employing gold nanoparticles as labels were extensively reported for diverse analytical applications in the literature in recent years [77–79]. Besides gold nanoparticles, silver nanoparticles are also widely used as labels in the construction of both immuno- and aptasensors [80,81]. Similarly, DNA probes are modified with different types of semiconductor nanoparticles such as ZnS, CdS and PbS in the fabrication of electrochemical DNA-based aptasensors [82–84]. Some of the above described nanomaterials have been only demonstrated with voltammetry detection methods, but they may find potential applications in the amperometric detection in the future.

Nanoparticles are composed of thousands of electrochemically active atoms. When biomolecules/analytes are labeled with nanoparticles, the loading of electroactive species on the electrode surface increases, and subsequently the sensitivity of the system is improved. Nanoparticles of gold, ZnS, CdS, PbS and silver have been explored in this regard [85–89]. However, many nanoparticles are electrochemically inactive within the potential range of aqueous electrolyte media or have redox potentials close to the solvent potential limit where the background signal may interfere. This drawback is associated with the intrinsic stability of nanoparticles in addition to the presence of stabilizer agents on nanosurfaces. Consequently, this drawback makes it difficult to detect nanoparticle labels directly using electrochemical methods. To overcome this problem, nanoparticles are dissolved in solution using a strong oxidizing agent and subsequently electrochemical measurements are performed [90]. Since the concentration of metallic ions is proportional to the amount of nanoparticles, hence it can be related to the concentration of biomolecules in the sample solution. Alternatively, in order to avoid the need for an additional step to dissolve nanoparticle labels for their detection, another strategy is to explore the catalytic property of nanoparticles and monitor the amount of nanoparticles on the electrode surface indirectly by the process they catalyze. In this approach, after performing the labeling process, biosensors are incubated with the solution of nanoparticle electrochemically active substrates. Under these conditions, the biosensor acts like an ultramicroelectrode array [91]. This strategy works well only in case of nanoparticle labels that have very high catalytic activity, such as Pd, Au, Pt, Ag-Au alloy and Pt alloy [92–96]. Since most of the nanomaterials are highly resistant to electrooxidation, a direct detection strategy by employing catalytic properties is an attractive alternative. For example, Wang *et al*., used Au/Ag/Au core/double shell nanoparticles (Au/Ag/Au NPs) as novel enzyme-mimetic labels for the development of a sandwich-type electrochemical immunosensor for SCCA. Au NPs decorated mercapto-functionalized graphene sheets (Au@SH-GS) were used as platform for the immobilization of primary antibody (Ab1), while Au/Ag/Au NPs were employed as the secondary antibody (Ab2) label. Due to the excellent electrocatalytic activity of Au/Ag/Au NPs towards the reduction of hydrogen peroxide, the designed immunosensor displayed an excellent analytical performance with good reproducibility, high selectivity and stability [97].Similarly, Lu *et al*., reported carbon nanotube/manganese dioxide (CNT–MnO_2_) composites as electrochemical tags for non-enzymatic signal amplification in immunosensing. The synthesized CNT–MnO_2_ composites showed good electrochemical activity, electrical conductivity and stability in a hydrogen peroxide environment. The electrochemical immunosensor demonstrated a linear response on a log-scale for AFP [98]. Li *et al*., developed a novel sandwich-type nonenzymatic electrochemical immunosensor for the cancer biomarker carbohydrate antigen 15–3. Hierarchical nanoporous PtFe alloys were used as carriers of the signaling antibody anti-CA15-3 in order to achieve signal amplification. The electrochemical signal of the immunosensor was based on the high electrocatalytic activity of PtFe alloy for the electro-oxidation of H_2_O_2_. Theimmunosensor showed good accuracy, stability and fabrication reproducibility [99]. Despite the many advancements, many nanopaticles having catalytic properties are yet to be explored.

The analytical performance of labeled affinity sensors based on antibodies and aptamers is strongly dependent on the pH of the buffer, nature of the buffer, temperature and salt concentration in the buffer. Each affinity-based sensor has a specific optimized experimental condition range, and any change in the experimental conditions and binding medium may affect the characteristics of biosensors. On the other hand, nanomaterials are also very sensitive to the dispersion medium and pH. Nanomaterials may aggregate or agglomerate at any given pH, and the presence of salts is also known to aggregate the nanoparticles. For example, silver and gold nanoparticles aggregate in the presence of salts of sodium, potassium and manganese. Phosphate buffer is the commonly used medium to perform the affinity based electrochemical biosensors. Researchers have also shown the adsorption of phosphate ions on the surface of nanomaterials. It is always very hard to find optimal experimental conditions and medium where both affinity interaction and nanomaterials retain their functionalities. Future research in this context may focus on the synthesis of nanomaterials that can retain the intrinsic properties under any experimental conditions, and their functions will be independent of the pH of the medium.

### 2.4. Nanomaterials as Enzyme Mimics

Recently, nanomaterials have attracted tremendous interest in enzyme mimetic research because of their several distinct properties, such as high surface area to volume ratio, abundance of reactive groups on their surface, unique optical properties and fascinating catalytic activity. To date, catalase-, oxidase-, peroxidase- or oxidase-like activities have been demonstrated for various types of nanomaterials [100]. The most commonly investigated nanomaterials are gold nanoparticles, Fe_3_O_4_ nanoparticles, sheet like-FeS nanostructures, single walled carbon nanotubes, graphene oxide, AgM nanoparticles, nanoceria and metallic nanocomposites [101]. These nanomaterial-based enzyme mimetics offer the advantages of low cost, high stability and sustained catalytic activities. As a consequence, biosensors based on these nanomaterials have been extensively employed for many applications in the bioassay, biotechnology and biomedical field. For example, many electrochemical biosensors based on the enzyme-like activity of silver, gold, graphene oxide and Fe_3_O_4_ for electrochemical detection of hydrogen peroxide, glucose and many other analytes have been reported in the literature [59,102–104]. However, nanomaterials do not have not real enzyme-like properties, as it is very difficult to regenerate the nanomaterial surface for subsequent measurement, limiting their applications in amperometric biosensors. Moreover, controlling the reactivity of nanomaterials against certain interfering molecules is a very difficult task. The reactivity of nanomaterials mainly relies on the functional groups of the analytes, and closely related interfering molecules share a very similar structure to the focus analyte. This reactivity may result in generation of signals even in the absence of analyte and produce false positive results. To replace enzymes for biosensing applications, it is highly desirable to design selective and specific nanomaterials to overcome the matrix interferences.

### 2.5. Composite Nanomaterials in Amperometric Biosensors

The nanomaterial hybrids made by combining nanostructures (nanoparticles, nanorods) exhibit properties of both materials, *i.e.*, the electronic structure and characteristics of the individual nanomaterials [105]. With appropriate design, these conjugates can be employed in electrochemical biosensors. Recently, such hybrid and composite nanomaterials have attracted great attraction in the field of electrochemical biosensors, along with other domains. These hybrid structures can serve as immobilization supports, signal amplifiers and electrochemical signal generating probe. For examples, Iron-silica composites have been used for biomolecule immobilization due to their better biocompatibility [106]. Other commonly used nanocomposites for immobilization purposes include magnetic nanoparticles-silica, Fe_3_O_4_-chitosan, PAni/PVA-AgNP and Pt/carbon nanotubes [107–110]. Similarly, composites of silver and gold nanoparticles have been explored to amplify the electrochemical signal as well as electrochemical signal generating probes in affinity-based sensors [111]. Pt and ZnOhave the potential to be used in combination with various types of nanomaterials to enhance the performance of amperometric biosensors [112]. It is anticipated that the combination of diverse properties of nanomaterials into a single composite structure will open new gateways for research in many fields

## 3. Conclusions

Nanomaterial-based amperometric biosensors present a novel and attractive paradigm in terms of new and augmented functionality that encompasses a wide variety of application in the analytical domain. This review provided a brief survey of the enormous variety of functions of nanomaterials, which are attributed to the unique physiochemical properties of the constituent nanomaterials, that could find potential applications in amperometric biosensors. Table 1 presents some of the examples of nanomaterial functionalities in electrochemical biosensors. Amperometric biosensors based on nanomaterials may offer various advantages for enhancing and superseding the capabilities of current analytical methodology by permitting rapid and highly accurate analysis. Despite much progress, this field is still new, and there are many points in the development of nanomaterial-based amperometric biosensors to be addressed such as: (a) many complex biological systems require specific physiological environments and a certain degree of biocompatibility, and the biosensor-integrated nanomaterial must fulfill this requirement; (b) it is highly desirable to find nanomaterials with sufficient binding sites for biomolecules; (c) the possibility to controllably tune the nanomaterials' properties for simultaneous analysis of various anlytes is yet to be explored; (d) although there are reports on the joint use of nanomaterials, most of nanomaterial combinations still need to be explored; and (e) the problem of nanomaterial aggregation in complex media should be resolved. Future research may focus on improving the above described parameters to further improve the analytical characteristic of nanomaterial-based amperometric biosensors.

## Figures and Tables

**Table 1.** Some of the examples of nanomaterials functions in electrochemical biosensors.

**Sr No**	**Functions**	**Nanomaterial Use**	**Type of Biosensor**	**Ref.**
1	Immobilization support	iron oxide–chitosan nanocomposite	Electrochemical enzymatic biosensor	[113]
2	Immobilization support	Gold nanoparticles and graphene	Electrochemical DNA biosensor	[114]
3	Immobilization support	Gold nanoparticles/polyaniline nanofibers	Electrochemical immunosensor	[115]
4	Immobilization support	Graphene nanoplatelet–titanate nanotube composite	Electrochemical enzymatic biosensor	[116]
5	Immobilization support	Graphene oxide and silver nanoparticles	Electrochemical enzymatic biosensor	[117]
6	Signal amplification	Silver nanoparticles	Electrochemical DNA biosensor	[118]
7	Signal amplification	Platinum nanotubes modified with polyamidoamine	Electrochemical DNA biosensor	[119]
8	Signal amplification	Gold nanoparticles	Electrochemical immunosensor	[120]
9	Signal amplification	Ferroferric oxide nanoparticle	Electrochemical immunosensor	[121]
10	Signal amplification	Graphene, platinum nanoparticles	Electrochemical immunosensor	[122]
11	Signal generating probe	Cu@Ag (Cu@Ag-CD) core–shell nanoparticles	Electrochemical immunosensor	[123]
12	Signal generating probe	Magnetic beads	Electrochemical immunosensor	[124]
13	Signal generating probe/mediator	PtCo Alloy and graphene	Electrochemical immunosensor	[125]
14	Improve electrical properties	Carboxygraphene	Enzymatic biosensors	[126]
15	Improve electrical properties	Nano gold/graphene	Electrochemical myoglobin biosensor	[127]
16	Enzyme mimics	Graphene oxide (ERGO)–silver nanoparticle	Hydrogen per oxide sensor	[128]
17	Enzyme mimics	MnO_2_	Hydrogen peroxide sensor	[129]
